# Supplementary material for: How, why and when are delayed (back-up) antibiotic prescriptions used in primary care? A realist review integrating concepts of uncertainty in healthcare
Source: BMC Public Health. 2024 Oct 14;24:2820. doi: 10.1186/s12889-024-20248-8 (PMC11476980; doi:10.1186/s12889-024-20248-8)
Supplement: Supplementary file 2 — Supplemental file 2. Document characteristics, source, use and nature of evidence. [file 12889_2024_20248_MOESM2_ESM.pdf]

Supplemental file 2

Document characteristics: source, use, and nature of evidence

Data Source	Use	Nature of source/description
Literature	Formal data	76 articles from Medline (from 1950), EMBASE (from 1947), Web of Science (from 1970), Cochrane Database of Systematic Reviews, and PsycINFO (from 1950). There were 28 articles from the UK; 15 from the USA; six from Australia; five from Ireland; four each from the Netherlands and New Zealand; three each from Norway, Canada and Spain; one each from Denmark, Ghana, Israel and Malta; one was undetermined. There were 50 empirical studies (including three quality improvement studies): 15 qualitative studies; 18 randomised trials; four other types of trials; nine observational; three quantitative; one mixed-methods. Twenty-three empirical studies were from the UK; six from the USA; five from Ireland; three each from Australia, Spain, and Norway; the remainder from New Zealand, the Netherlands, Italy, Canada, Denmark, Israel, Ghana, Malta. We included nine literature reviews and 17 commentary articles.<br>Included articles are listed alphabetically below.
Interviews	Formal data	Excerpts from 41 interviews conducted as part of wider 'STEP-UP' study (https://www.expmedndm.ox.ac.uk/step-up) to explore experiences of Clinical Commissioning Group and general practice professionals in implementing antimicrobial stewardship interventions.[1] Only excerpts focused on participants' discussion of DP use were included in this review.
Wider STEP-UP team and advisory group	Feedback, advice and sense-making	Discussion and feedback from four face-to-face meetings (three in the regular wider STEP-UP team meetings and one as part of the advisory group meeting). In addition to the multi-professional and interdisciplinary expertise of the wider STEP-UP team, the advisory panel had representation from a lay member, commissioner, national policy maker and an epidemiologist.

Included articles

Agnew, J. *et al.* (2013) "Delayed Prescribing of Antibiotics for Respiratory Tract Infections: Use of Information Leaflets," *Irish Medical Journal*, 106(8), pp. 243–4.

Alonso-Coello, P., Llor, C. and de la Poza Abad, M. (2016) "Mixed Diagnoses and Mixed Messages—Reply," *JAMA Internal Medicine*, 176(5).

Andrews, T. *et al.* (2012) "Interventions to influence consulting and antibiotic use for acute respiratory tract infections in children: A systematic review and Meta-Analysis," *PLoS ONE*, 7(1).

Arnold, S.R. and Straus, S.E. (2005) "Interventions to improve antibiotic prescribing practices in ambulatory care," *Cochrane Database of Systematic Reviews*, 15(16).

Arroll, B. *et al.* (2002) "Delayed antibiotic prescriptions: what are the experiences and attitudes of physicians and patients?," *Journal of Family Practice*, 51(11), pp. 954–959.

Arroll, B. *et al.* (2003) "Delayed prescriptions Can reduce antibiotic use in acute respiratory infections," *BMJ*, 327, pp. 1361–2.

Arroll, B., Kenealy, T. and Kerse, N. (2002) “Do Delayed Prescriptions Reduce the Use of Antibiotics for the Common Cold? A Single-Blind Controlled Trial,” *Journal of Family Practice*, 51(4), pp. 324–328.

Arroll, B., Kenealy, T. and Kerse, N. (2003) “Do delayed prescriptions reduce antibiotic use in respiratory tract infections? A systematic review ,” *British Journal General Practice*, 53(496), pp. 1–7.

Ashdown, H.F. *et al.* (2016) “Prescribing antibiotics to ‘at-risk’ children with influenza-like illness in primary care: Qualitative study,” *BMJ Open*, 6(6).

Boiko, O. *et al.* (2020) “Risks of use and non-use of antibiotics in primary care: qualitative study of prescribers’ views,” *BMJ Open*, 10(10), p. e038851.

Borek, A.J. *et al.* (2020) “Social and contextual influences on antibiotic prescribing and antimicrobial stewardship: A qualitative study with clinical commissioning group and general practice professionals,” *Antibiotics*, 9(12), pp. 1–15.

Borek, A.J. *et al.* (2021) “Implementing interventions to reduce antibiotic use: a qualitative study in high-prescribing practices,” *BMC Family Practice*, 22(1).

Bozzella, M.J. *et al.* (2018) “From paper to practice: Strategies for improving antibiotic stewardship in the pediatric ambulatory setting,” *Current Problems in Pediatric and Adolescent Health Care*, 48(11), pp. 289–305.

Cals, J.W.L. *et al.* (2010) “Point-of-care c-reactive protein testing and antibiotic prescribing for respiratory tract infections: A randomized controlled trial,” *Annals of Family Medicine*, 8(2), pp. 124–133.

Cates, C. (2003) “Delayed prescriptions in primary care,” *British Journal of General Practice*, 53(496), pp. 836–837.

Cimolai, N. (2020) “Delayed Antibiotic Prescribing in the Outpatient Setting,” *Journal of Pharmacy Practice*, 33(6), pp. 736–737.

Cooper, E. *et al.* (2020) “Diagnosis and Management of UTI in Primary Care Settings—A Qualitative Study to Inform a Diagnostic Quick Reference Tool for Women Under 65 Years,” *Antibiotics*, 9(9), p. 581.

Couchman, G.R., Rascoe, T.G. and Forjuoh, S.N. (2000) “Back-up Antibiotic Prescriptions for Common Respiratory Symptoms ,” *Journal of Family Practice*, 49(10).

Dallas, A. *et al.* (2020) “Delayed prescribing of antibiotics for acute respiratory infections by GP registrars: a qualitative study,” *Family Practice*, 37(3), pp. 406–411.

Damoiseaux, R. (2004) “Delayed prescriptions — not a good option for infants,” *The British Journal of General Practice*, 54(498), p. 58.

Dharod, A. (2016) “Delayed Prescriptions for Reducing Antibiotic Use ,” *Journal of Clinical Outcomes Management*, 23(3).

Dowell, J *et al.* (2001) *A randomised controlled trial of delayed antibiotic prescribing as a strategy for managing uncomplicated respiratory tract infection in primary care*.

Duane, S. *et al.* (2016) “Exploring experiences of delayed prescribing and symptomatic treatment for urinary tract infections among general practitioners and patients in ambulatory care: A qualitative study,” *Antibiotics*, 5(3).

Flynn, M. and Hooper, G. (2020) “Antimicrobial stewardship though FeverPAIN score: Successes and challenges in secondary care,” *Clinical Infection in Practice*, 7–8, p. 100024.

Francis, N.A. *et al.* (2012) “Delayed antibiotic prescribing and associated antibiotic consumption in adults with acute cough,” *British Journal of General Practice*, 62(602), pp. e639–e646.

Ghebrehewet, S. *et al.* (2020) “Implementation of a Delayed Prescribing Model to Reduce Antibiotic Prescribing for Suspected Upper Respiratory Tract Infections in a Hospital Outpatient Department, Ghana,” *Antibiotics*, 9(11), p. 773.

Hansen, P. (2016) *Antibiotic Resistance: Use of Delayed Prescriptions for Viral Syndromes in Urgent Care*. Available at: http://scholarship.shu.edu/final-projects/13.

Harris, D.J. (2013) “Initiatives to improve appropriate antibiotic prescribing in primary care,” *Journal of Antimicrobial Chemotherapy*, 68(11), pp. 2424–2427.

Hay, A.D. (2004) “Review: delaying a prescription reduces antibiotic use in upper respiratory tract infections,” *Evidence-Based Medicine*, 9(4).

Hay, A.D. *et al.* (2019) “Anaesthetic–analgesic ear drops to reduce antibiotic consumption in children with acute otitis media: the CEDAR RCT,” *Health Technology Assessment*, 23(34), pp. 1–48.

Hayes, M., Faherty, A. and Hannon, D. (2013) “Delayed prescriptions: attitudes and experiences of General Practitioners in the Midwest,” *Irish Medical Journal* [Preprint].

Høye, S., Frich, J.C. and Lindbæk, M. (2011) “Use and feasibility of delayed prescribing for respiratory tract infections: A questionnaire survey,” *BMC Family Practice*, 12(34).

Høye, S., Frich, J.C. and Lindbœk, M. (2010) “Delayed prescribing for upper respiratory tract infections: A qualitative study of GPs’ views and experiences,” *British Journal of General Practice*, 60(581), pp. 907–912.

Høye, S., Gjelstad, S. and Lindbæk, M. (2013) “Effects on antibiotic dispensing rates of interventions to promote delayed prescribing for respiratory tract infections in primary care,” *British Journal of General Practice*, 63(616), pp. e777–e786.

Hughes, A. *et al.* (2016) *Evaluating a point-of-care C-reactive protein test to support antibiotic prescribing decisions in a general practice-a-point-of-care-c-reactive-protein-test-to-support-antibiotic-prescribing-decisions-in-a*, *Clinical Pharmacist*.

Ivers, N., Arroll, B. and Allan, M. (2011) “Delayed antibiotic prescriptions for URTIs,” *Canadian Family Physician*, 57(11), p. 1287.

Johnson, J.R. (2007) “Wait-and-See Prescription for Acute Otitis Media,” *JAMA*, 297(2), pp. 152–153.

Kavanagh, K.E. *et al.* (2011) “A pilot study of the use of near-patient C-Reactive Protein testing in the treatment of adult respiratory tract infections in one Irish general practice,” *BMC Family Practice*, 12.

Lacy, S.M. (2007) “Writing a wait-and-see prescription for the treatment of acute otitis media may decrease the use of antibiotics,” *The Journal of Pediatrics*, 150(3), p. 319.

de La Poza Abad, M. *et al.* (2016) “Prescription strategies in acute uncomplicated respiratory infections a randomized clinical trial,” *JAMA Internal Medicine*, 176(1), pp. 21–29.

de La Poza Abad, M. *et al.* (2019) “Use of delayed antibiotic prescription in primary care: A cross-sectional study,” *BMC Family Practice*, 20(1).

Linder, J.A. and Friedberg, M.W. (2016) “Mixed Diagnoses and Mixed Messages,” *JAMA Internal Medicine*, 176(5), pp. 718–719.

Link, T.L. *et al.* (2016) “Reducing inappropriate antibiotic prescribing for adults with acute bronchitis in an urgent care setting a quality improvement initiative,” *Advanced Emergency Nursing Journal*, 38(4), pp. 327–335.

Little, P. *et al.* (1997) "Open randomised trial of prescribing strategies in managing sore throat," *BMJ*, 314(722).

Little, P. (2001) "Pragmatic randomised controlled trial of two prescribing strategies for childhood acute otitis media," *BMJ*, 322, pp. 336–342.

Little, P. (2005) "Delayed prescribing of antibiotics for upper respiratory tract infection," *BMJ*, 331(301).

Little, P. *et al.* (2005) "Information Leaflet and Antibiotic Prescribing Strategies for Acute Lower Respiratory Tract Infection A Randomized Controlled Trial," *JAMA*, 293(24), pp. 3029–3035.

Little, P. (2006) *Delayed Prescribing-A Sensible Approach to the Management of Acute Otitis Media*.

Little, P. *et al.* (2009) "Dipsticks and diagnostic algorithms in urinary tract infection: Development and validation, randomised trial, economic analysis, observational cohort and qualitative study," *Health Technology Assessment*, 13(19).

Little, P. *et al.* (2010) "Effectiveness of five different approaches in management of urinary tract infection: Randomised controlled trial," *BMJ (Online)*, 340, p. c199.

Little, P. *et al.* (2013) "Clinical score and rapid antigen detection test to guide antibiotic use for sore throats: Randomised controlled trial of PRISM (primary care streptococcal management)," *BMJ (Online)*, 347.

Little, P. *et al.* (2014) "Delayed antibiotic prescribing strategies for respiratory tract infections in primary care: Pragmatic, factorial, randomised controlled trial," *BMJ (Online)*, 348, p. g1606.

Little, P. *et al.* (2017) "Antibiotic prescription strategies and adverse outcome for uncomplicated lower respiratory tract infections: Prospective cough complication cohort (3C) study," *BMJ (Online)*, 357, p. j2418.

Little, P. (2020) "Delayed Antibiotic Prescriptions," *JAMA*, 324(13), pp. 1352–1353.

Lum, E.P.M. *et al.* (2018) "Antibiotic prescribing in primary healthcare: Dominant factors and trade-offs in decision-making," *Infection, Disease and Health*, 23(2), pp. 74–86.

McCullough, A.R. and Glasziou, P.P. (2016) "Delayed antibiotic prescribing strategies-time to implement?," *JAMA Internal Medicine*. American Medical Association, pp. 29–30.

McDonagh, M. *et al.* (2016) *Improving Antibiotic Prescribing for Uncomplicated Acute Respiratory Tract Infections*.

Moore, M. *et al.* (2009) "Effect of antibiotic prescribing strategies and an information leaflet on longer-term reconsultation for acute lower respiratory tract infection," *British Journal of General Practice*, 59(567), pp. 728–734.

Morrell, L. *et al.* (2020) "Delayed Antibiotic Prescription by General Practitioners in the UK: A Stated-Choice Study," *Antibiotics*, 9(9).

Peters, S. *et al.* (2011) "Managing self-limiting respiratory tract infections: A qualitative study of the usefulness of the delayed prescribing strategy," *British Journal of General Practice*, 61(590), pp. e579–e589.

Pshetizky, Y., Naimer, S. and Shvartzman, P. (2003) "Acute otitis media - A brief explanation to parents and antibiotic use," *Family Practice*, 20(4), pp. 417–419.

Raft, C.F. *et al.* (2017) "Delayed antibiotic prescription for upper respiratory tract infections in children under primary care: Physicians' views," *European Journal of General Practice*, 23(1), pp. 191–196.

Redmond, N.M. *et al.* (2018) “Impact of antibiotics for children presenting to general practice with cough on adverse outcomes: Secondary analysis from a multicentre prospective cohort study,” *British Journal of General Practice*, 68(675), pp. e682–e693.

Rowe, T. and Linder, J.A. (2019) “Novel approaches to decrease inappropriate ambulatory antibiotic use,” *Expert Review of Anti-infective Therapy*, 17(7), pp. 511–521.

Rowe, T. and Linder, J.A. (2020a) “Delayed Antibiotic Prescriptions in Ambulatory Care: Reconsidering a Problematic Practice,” *JAMA - Journal of the American Medical Association*. American Medical Association, pp. 1779–1780.

Rowe, T. and Linder, J.A. (2020b) “Delayed Antibiotic Prescriptions—Reply,” *JAMA*, 324(13), p. 1353.

Ryves, R. *et al.* (2016) “Understanding the delayed prescribing of antibiotics for respiratory tract infection in primary care: A qualitative analysis,” *BMJ Open*, 6(11).

Saliba-Gustafsson, E.A. *et al.* (2019) “General practitioners’ perceptions of delayed antibiotic prescription for respiratory tract infections: A phenomenographic study,” *PLOS ONE*, 14(11), p. e0225506.

Sargent, L. *et al.* (2016) “Is Australia ready to implement delayed prescribing in primary care? A review of the evidence,” *Australian Family Physician*, 45(9), pp. 688–90.

Sargent, L. *et al.* (2017) “Using theory to explore facilitators and barriers to delayed prescribing in Australia: a qualitative study using the Theoretical Domains Framework and the Behaviour Change Wheel,” *BMC Family Practice*, 18(1).

Septimus, E.J. *et al.* (2017) “Extended-Release Guaifenesin/Pseudoephedrine Hydrochloride for Symptom Relief in Support of a Wait-and-See Approach for the Treatment of Acute Upper Respiratory Tract Infections: A Randomized, Double-Blind, Placebo-Controlled Study,” *Current Therapeutic Research - Clinical and Experimental*, 84, pp. 54–61.

Siegel, R.M. *et al.* (2003) “Treatment of Otitis Media With Observation and a Safety-Net Antibiotic Prescription,” *Pediatrics*, 112(3), pp. 527–531. Available at: www.aappublications.org/news.

Spiro, D.M. *et al.* (2006) *Wait-and-See Prescription for the Treatment of Acute Otitis Media A Randomized Controlled Trial*.

Spurling, G.K.P. *et al.* (2017) “Delayed antibiotic prescriptions for respiratory infections,” *Cochrane Database of Systematic Reviews* [Preprint]. John Wiley and Sons Ltd.

Thoolen, B., de Ridder, D. and van Lensvelt-Mulders, G. (2012) “Patient-oriented interventions to improve antibiotic prescribing practices in respiratory tract infections: A meta-analysis,” *Health Psychology Review*, 6(1), pp. 92–112.

Vellinga, A. *et al.* (2016) “Intervention to improve the quality of antimicrobial prescribing for urinary tract infection: A cluster randomized trial,” *CMAJ*, 188(2), pp. 108–115.

Vervloet, M. *et al.* (2016) “Reducing antibiotic prescriptions for respiratory tract infections in family practice: Results of a cluster randomized controlled trial evaluating a multifaceted peer-group-based intervention,” *npj Primary Care Respiratory Medicine*, 26.

REFERENCES

1 Borek AJ, Anthierens S, Allison R, *et al.* Social and contextual influences on antibiotic prescribing and antimicrobial stewardship: A qualitative study with clinical commissioning group and general practice professionals. *Antibiotics* 2020;**9**:1–15.
