# Supplementary material for: How, why and when are delayed (back-up) antibiotic prescriptions used in primary care? A realist review integrating concepts of uncertainty in healthcare
Source: BMC Public Health. 2024 Oct 14;24:2820. doi: 10.1186/s12889-024-20248-8 (PMC11476980; doi:10.1186/s12889-024-20248-8)
Supplement: Supplementary file 3 — Supplemental file 3. Categorising contexts, mechanisms and outcomes. [file 12889_2024_20248_MOESM3_ESM.pdf]

Supplemental file 3

Categorising contexts, mechanisms and outcomes

The concepts of context, mechanism and outcome can be challenging to define, even among realist experts, and the same thing can be a context in one circumstance, a mechanism in another, and an outcome in yet another—what is important is how these explain how and why something happens.(1) We therefore took a pragmatic approach to classifying contexts and mechanisms to produce four outcomes: immediate antibiotics; delayed prescription (DP) instead of immediate antibiotics; DP instead of no antibiotics; and no antibiotics. We identified 'contexts' and 'mechanisms' labels where the former were directly observable or perceived and the latter were mainly invisible, e.g. beliefs. Three researchers (AC, MMc, GW) organised these into potential context-mechanism-outcome configurations (CMOCs). We designated items as 'context' or 'mechanism' when we found evidence that their positions in the CMOC could be swapped/changed.(1)

References

1. Westhorp G. Understanding Mechanisms in Realist Evaluation and Research. In: Emmel N, Greenhalgh J, Manzano A, Monaghan M, Dalkin S (eds.) *Doing Realist Research*. SAGE Publications Ltd; 2019. p. 41–57.
