# Supplementary figures and images for: How, why and when are delayed (back-up) antibiotic prescriptions used in primary care? A realist review integrating concepts of uncertainty in healthcare
Source: BMC Public Health. 2024 Oct 14;24:2820. doi: 10.1186/s12889-024-20248-8 (PMC11476980; doi:10.1186/s12889-024-20248-8)

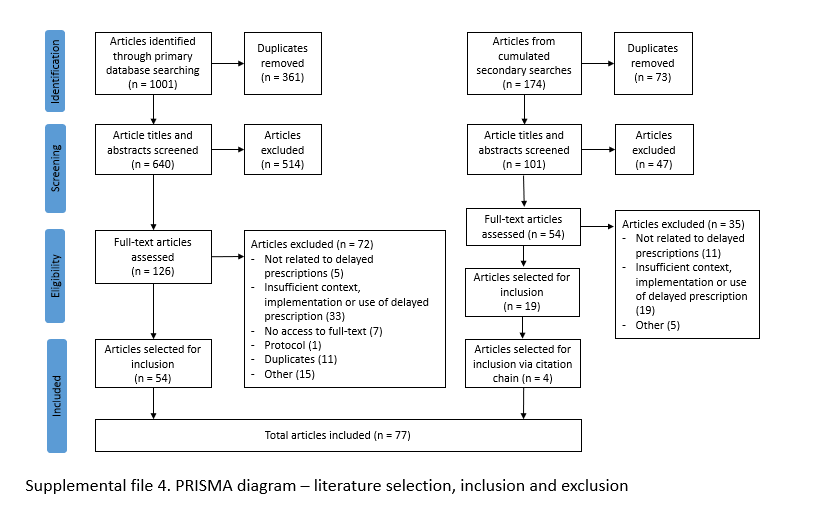

Supplement: Supplementary file 4 — Supplemental file 4. Literature selection, inclusion and exclusion. [file 12889_2024_20248_MOESM4_ESM.jpg]

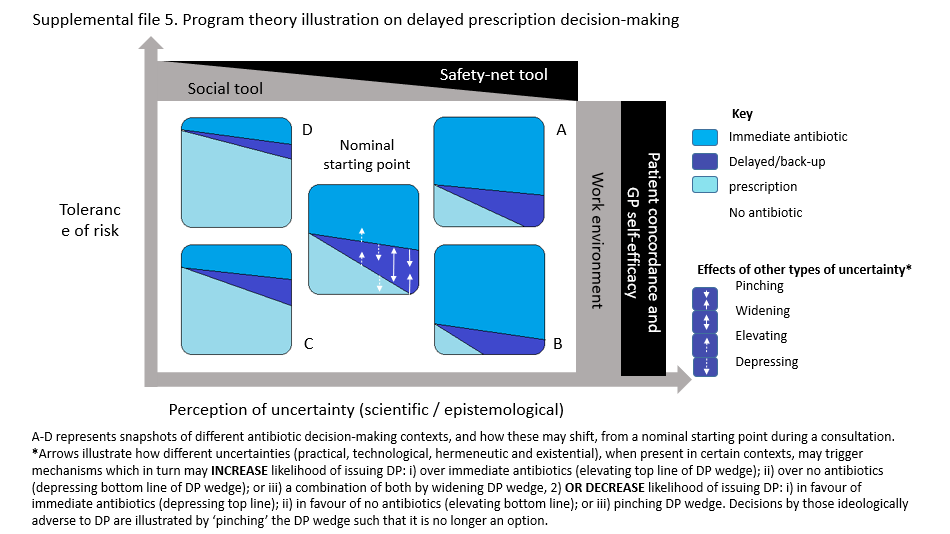

Supplement: Supplementary file 5 — Supplemental file 5. Program theory on delayed prescription decision-making. [file 12889_2024_20248_MOESM5_ESM.jpg]
